# The management of turn transition in signed interaction through the lens of overlaps

**DOI:** 10.3389/fpsyg.2015.00741

**Published:** 2015-06-18

**Authors:** Simone Girard-Groeber

**Affiliations:** ^1^Departement Heilpädagogische Lehrberufe, University of Applied Sciences of Special Needs EducationZurich, Switzerland; ^2^Centre de Linguistique Appliquée, University of NeuchâtelNeuchâtel, Switzerland

**Keywords:** conversation analysis, sign language interactions, overlap, simultaneous signing, turn transition, Swiss German Sign Language (*Deutschschweizerische Gebärdensprache*, DSGS)

## Abstract

There have been relatively few studies on sign language interaction carried out within the framework of conversation analysis (CA). Therefore, questions remain open about how the basic building blocks of social interaction such as turn, turn construction unit (TCU) and turn transition relevance place (TRP) can be understood and analyzed in sign language interaction. Recent studies have shown that signers regularly fine-tune their turn-beginnings to potential completion points of turns (Groeber, 2014; Groeber and Pochon-Berger, 2014; De Vos et al., 2015). Moreover, signers deploy practices for overlap resolution as in spoken interaction (McCleary and Leite, 2013). While these studies have highlighted the signers' orientation to the “one-at-a-time” principle described by Sacks et al. (1974), the present article adds to this line of research by investigating in more detail those sequential environments where overlaps occur. The contribution provides an overview of different types of overlap with a focus of the overlap's onset with regard to a current signer's turn. On the basis of a 33-min video-recording of a multi-party interaction between 4 female signers in Swiss German Sign Language (DSGS), the paper provides evidence for the orderliness of overlapping signing. Furthermore, the contribution demonstrates how participants collaborate in the situated construction of turns as a dynamic and emergent gestalt and how they interactionally achieve turn transition. Thereby the study adds to recent research in spoken and in signed interaction that proposes to rethink turn boundaries and turn transition as flexible and interactionally achieved.

## Introduction

The precursory work of Sacks et al. (1974) on the machinery of turn-taking in conversation has built the foundation for the conversation analytic tradition. Their ground-breaking paper described how participants in conversation finely coordinate their *turns-at-talk* by minimizing both gaps and overlaps during the transition from a current speaker to a next speaker. Subsequent research in Conversation Analysis (henceforth: CA) has further demonstrated the robustness of Sacks et al.'s (1974) model of turn-taking being achieved on the principle of “one-at-a-time” in interactions involving other languages than English (e.g., Stivers et al., 2009), different contexts (informal and institutional) as well as diverse types of speakers (e.g., L1 and L2 speakers for example by Carroll, 2000; Gardner, 2007).

However, research that pointed to an increased amount of simultaneous talk or of pauses between turn transitions, has also questioned the turn-taking *machinery* as a universal model (as e.g., Tannen, 1984 or Lehtonen and Sajavaara, 1985, cited by Gardner et al., 2009). It was suggested that linguistic and cultural aspects are the reason for such a variation between different turn-taking systems. The present study contributes to this issue by investigating the sequential organization of social interaction in a *signed language*. Signed languages make use of the sequential and simultaneous combination of spatio-visual resources, which can be more or less conventionalized among a specific sign language community. To date there have been relatively few studies on sign language interaction carried out within the framework of CA. Interestingly however, most of the researchers working on signed interaction have to some extent discussed the issue of overlapping signing (e.g., Martinez, 1995; McIlvenny, 1995; Coates and Sutton-Spence, 2001; Lackner, 2009; McCleary and Leite, 2013; De Vos et al., 2015). Some researchers highlight the fact that signed interaction presents a dense occurrence of simultaneous signing, sometimes explicitly questioning the relevance of the one-at-a-time model proposed by Sacks et al. (1974) for signed interaction (Coates and Sutton-Spence, 2001; Lackner, 2009). Other researchers rather emphasize the fact that signers finely coordinate their turn beginnings to potential completion points of current signers (McCleary and Leite, 2013; De Vos et al., 2015).

With this paper I intend to add to this topic by a detailed investigation of those instances in signed interaction where participants actually overlap each other's turns, by focusing specifically on the sequential environment of these overlaps (i.e., onset of the overlap at the beginning, midst or end of the current speaker's turn). This type of investigation sheds light on two issues. First, the analysis of the sequential moment of overlaps aims at revealing whether they present an orderly organization, as it has been shown for overlap in spoken language interaction (cf. Section Overlaps in Spoken and Signed Interaction). Second, the analysis of overlapping signing provides insight into how four participants of a signed language conversation accomplish the actual transitions from one signer to the other. On the basis of a 33-min video-recording of a multi-party interaction with 4 female signers in Swiss German Sign Language (DSGS), the paper shows that (1) signers overlap each other's turns regularly *within possible transition spaces* and not in the midst of syntactic constructions, revealing therefore the same orderliness of overlap as in spoken language interaction; (2) signers actively accomplish smooth transitions between the current and the next signer, collaborating thereby in a situated and collaborative construction of turns. The findings add to recent research in spoken and in signed interaction that proposes to conceive turn boundaries as flexible and interactionally achieved.

I start with providing some details with regard to turn-taking and overlap in signed languages (Section Research on Turn-Taking and Overlap in Sign Language), presenting my conception of turn and further detailing the issue of this study. Then I present the methodology and procedure I followed for the current study (Section Method), specifying the annotation practice and the established categories for analysis. In Section Sequential Environments of Overlapping Signing, I present the results on different types of overlaps before I discuss these findings in Section Discussion.

## Research on turn-taking and overlap in sign language

### The lexical unit in sign language

**Figure 1:**
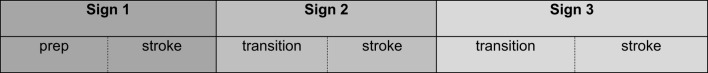
**Segmentation of signs including preparation and transition phases**.

**Figure 2:**
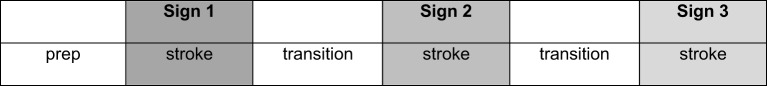
**Segmentation of signs excluding preparation and transition phases**.

The lexical unit in sign language is the manual *sign*, which consists of a distinct combination of four sublexical manual parameters, namely handshape, location (in the signing space), orientation (of the palms) and movement (Boyes Braem, 1990). Non-manual features such as gaze direction or facial expression can have distinctive effect on the lexical level. The sign unfolds sequentially in three basic phases, the *preparation phase* (i.e., hands are brought from rest position to the initial location, orientation and handshape), the *stroke* or *independent hold* (i.e., the proper semantic deployment of the sign) and the *retraction* (i.e., after full deployment the hands are brought back to rest position) (Kita et al., 1998). When annotating signed languages, researchers segment lexical signs in two different ways: either they consider end of one sign to be the start of the next sign (i.e., there is no gap between two signs, the transition from one sign to the other is assigned to the second sign; cf. Figure 1), or the start of a sign corresponds to the full deployment of the manual parameters handshape, location and orientation and ends with the end of the stroke, while transition phases are not part of the sign (i.e., there is a gap between two signs; cf. Figure 2) (cf. Hanke et al., 2012).

### The *turn* and *turn construction unit* in conversation analysis and sign language research

#### Turn and TCU in classic CA

In spoken interaction, the beginning and the ending of a participant's contribution represent the delimitation of a *turn*. Each turn can further be built by one or more *turn construction units* (Sacks et al., 1974; henceforth: TCU)^1^. Traditionally, TCUs have been defined as grammatical segments of talk, i.e., lexical, phrasal, clausal or sentential constructions, which are *interactionally relevant*. By interactionally relevant it is meant that participants orient to these units as possibly complete units that end in *transition relevance places* (Sacks et al., 1974; henceforth: TRP), i.e., places where the transition to a next speaker becomes *possible*. This transition is, however, not automatic, because co-participants might not take a next turn and current speakers can extend their turns after the possible completion (with new TCUs or by extending the previous TCU; Sacks et al., 1974; Schegloff, 1996). It is important to note as well that each TCU does not end in a TRP, which is the case in multi-unit turns such as story-tellings. In such cases the TRP can be blocked, for example, by lexical or pragmatic devices (Selting, 2000), e.g., the negotiation of a multi-unit turn through a preface.

The determination of TCUs has given rise to much debate within the field of CA, about, for example, the relative importance of syntactic and prosodic resources for the indication of completeness of a TCU (e.g., Ford et al., 1996; Schegloff, 1996; Selting, 1996, 2000). The importance of syntactic completion has been demonstrated by Selting (1996). She showed that participants finely time their recipiency tokens or early turn starts to possible syntactic boundaries of a current speaker's turn, showing thereby that they rely on their understanding of syntactic boundaries for the management of turn transitions. A consequence of this is for example that turn-final tag questions (in German “ne”; Selting, 1996, p. 363) are regularly overlapped with next speaker's turn start. However, it is not only syntax that determines whether a TCU may be complete or not. According to Selting, the TCU is a *linguistic unit* constructed with syntactic and prosodic resources, and it is considered linguistically complete in a given sequential and pragmatic context (Selting, 1996, 2000). She states that “*[i]t is the interplay of syntax and prosody that constitutes and delimits TCUs in general*.” (2000, p. 489). Put simply, a participant can design a complete syntactic construction but prosodic resources may indicate that there is more to come (cf. rush-through by Schegloff, 1982)—in which case it is not a TCU. In a similar manner, already Local and Kelly (1986) have shown how participants use features such as pitch, loudness and tempo or even glottal stops before a silence for indicating that e.g., a turn is not yet complete (projection of turn continuation) or that an overlapped turn was not complete and will be taken up again after the overlap (Local, 1992).

While TCUs (and turns) are traditionally conceived of as linguistic units, several researchers put into question a logocentric definition of turns-at-talk, where the *vocal production* of *grammatical units* appears to be a basic criterion for being considered part of a turn (cf. recent contributions in Rasmussen et al., 2014). Keevallik (2014) for example proposed an analysis of (non-linguistic) vocalizations as TCUs in their own right (cf. also Goodwin et al., 2000, on the use of nonsense syllables as TCUs or Ford et al., 2012). In a similar vein, an increasing quantity of research on bodily practices shows the relevance of these resources in the construction of “turns” and in the recipient's orientation to these bodily practices as being constitutive for the situated accomplishment of activities (e.g., Goodwin, 2000; Mondada, 2007; Oloff, 2013). In sum, these studies emphasize the fact that social interaction is based on the participants' accomplishment of *actions* by means of ensembles of resources such as speech, gesture, posture and gaze (Kääntä, 2010, proposes to speak of *turns-of-actions*). Within such a perspective, the delimitation of TCUs becomes less important an issue, because the focus of analysis is not on linguistic constructions but on the practices for the organization of activities (Ford et al., 1996).

#### Turn and TCU in sign language research

Research on signed languages has tried to adopt and adapt the basic notions of conversation analytic research to the situated organization of signed dialogs or social interaction. However, detailed discussions of how the *turn* and the TCU have to be conceived of in signed interaction are still scarce (but see McCleary and Leite, 2013; De Vos et al., 2015). The major challenge for the conception of turns and TCUs in signed languages arises from the fundamental multimodality of signed discourse on the one hand, and the continuous use of lexical, semi-lexical and gestural resources as basic building blocks of meaning construction (Liddell, 2003,^2^; König et al., 2012) on the other hand. This resonates very clearly with the current discussions on turn and TCU by those researchers who put into question a logocentric definition of these basic building blocks (cf. Section Turn and TCU in classic CA).

A first fundamental issue in research on signed interaction is how to define the beginning and the end of a turn. Lackner (2009) considered that the lowering and lifting of the forearms constitute turn boundaries. This entails for example that when a participant is holding a sign, he is not yet relinquishing his turn (cf. also Baker, 1977). De Vos et al. (2015) however delineate turns with respect to their stroke phases, excluding therefore the so-called non-verbal movements (holds, preparation phases and retraction phases). This delineation is based on a study that looked at turn transition times with three different phonetic measures (sign-naïve turn boundaries, i.e., transition is measured by considering preparation, stroke, hold and retraction phases; stroke-to-stroke turn boundaries, i.e., the transition is calculated by considering the time between two strokes; and a measure of transition between the next signer's preparation of his first sign in relation to the end of the stroke of the current signer's last sign). They further calculated the latency of these transitions and observed that for the *stroke-to-stroke turn boundary*, transition times were within cultural variation from spoken languages (i.e., 229 ms; Stivers et al., 2009). On the basis of this result they suggest that turns have to be delineated with respect to their strokes, whereas preparation, hold and retraction phases are excluded from the turn. In a study on Brazilian Sign Language, McCleary and Leite (2013) do not explicitly state their delineation of turns. Their analyses show, however, how a current signer self-interrupts his signing in response to gestural movements by an incipient signer, namely a self-groom and a palm-up gesture. Hence, the current signer is clearly orienting to these *non-verbal* resources as constituting overlapping signing that has to be resolved (even if it is not phonological, propositional or lexical). This would be an argument to consider such non-lexical resources as proper parts of signed turns.

In sum, the issue of defining turn boundaries in signed languages traces back to the question of whether non-verbal movements, i.e., preparation phases, holds and retraction phases as well as gestural elements such as palm-ups or even self-grooms should be considered as being part of the turn or not. In the present article turns are delineated in the following way:

1. The beginning of the *turn* includes the preparation phase of the turn-initial sign. The preparation phase of a turn-initial sign is comparable to a hearable inbreath in spoken interaction, described as a *pre-beginning* element (Schegloff, 1996). Pre-beginning elements are non-lexical elements such as an inbreath, coughs or gestures “*which can serve to initiate a turn, while not yet initiating a TCU within it*.” (Schegloff, 1996, p. 93). In a similar vein, Lindström (2006) proposes a differentiation between turn and TCU by considering *presegments* (e.g., pre-beginnings) and post-completions (e.g., *huh*) as not being part of the core TCU, but as a part of the possible turn. Against the background of this, I maintain a differentiation between *turns* as participant's overall contributions including resources beyond grammar, and the grammatical units that such turns (can) host.
2. The end of the *stroke of a potentially last sign* builds a first TRP, constituting also the end of the core grammatical unit (De Vos et al., 2015). However, I do not consider that the end of the linguistically built unit is automatically the end of the turn. Non-verbal movements that follow this stroke are considered as being part of the *turn*, even if they are not part of the grammatical unit (or the TCU). Consequently, holds after the stroke of the potentially last sign as well as additional gestural elements such as palm-up gestures are considered as being part of the turn. These additional elements extend the turn and finally propose a new TRP (cf. Lindström, 2006 on post-completers).

### The turn-taking machinery in signed languages

Preliminary and rather detailed observations on turn-taking in sign language can be found in Baker (1977), proposing an account for turn transition between signers based on the sending and interpreting of “signals” for turn regulation. Based on conversations between two dyads of deaf signers in a semi-experimental setting, she provides an overview of *initiation regulators, continuation regulators*, and *shift regulators*. The *initiation regulators* comprise those conducts that the incipient signer adopts for displaying that he will initiate a turn. The most fundamental initiation regulator is moving the hands out of *rest position* (in rest position the speaker's hands are relaxed e.g., on table or legs), providing a first visual index that the participant launches a turn. In case of absence of mutual gaze between participants, the incipient signer uses an *attention-getting device* (he waves his hand in the visual field of the addressee, taps on the table or the shoulder of the addressee) in order to establish recipiency. Baker (1977) attaches great importance to the establishment of mutual gaze by stating that the “*speaker cannot initiate a turn until the desired addressee looks at the potential speaker*” (Baker, 1977, p. 221). As already Coates and Sutton-Spence (2001) point out however, it seems that incipient signers regularly launch a turn even without previously established mutual gaze and without explicit attention-getting devices. Resources for this are restarts and holds of sign beginnings, allowing for a smooth turn beginning (cf. Goodwin, 1980 for restarts in spoken interaction). *Continuation regulators* refer to those “signals” that manifest that the signer will continue past a first “*information package*” or after a short pause (Baker, 1977, p. 218). According to Baker, not gazing at the co-participant, speeding up the signing and not returning to rest position as well as holding/freezing the last sign are regulators enabling the current signer to continue. This implies that if a speaker is not willing to abandon his turn, he can either “fill the pause” “*with small movements that indicate planning what next to say or by holding the final position of the last sign*” without gazing at the co-participant (Baker, 1977, p. 227). Finally, *shift regulators* are a set of behaviors used by the current or the incipient signer for signaling that the turn goes over to a next signer. Shift regulators by the incipient signer are understood as signals he deploys in overlap with the current signer's turn. Baker notes that e.g., increased size and quantity of head nods, palm-up gestures or gaze withdrawals are indicating that a co-participant attempts to shift from recipient status to signer status. The current signer's fundamental shift regulator is his gaze toward the potential next signer, as well as a diminution of the sign rate and the return to rest position (see also McIlvenny, 1995; McCleary and Leite, 2013 on overlap). Moreover, according to Baker, the end of a turn is signaled by returning the hands to rest position or by holding the last sign while gazing at the co-participant (cf. also Lackner, 2009)^3^.

### Overlaps in spoken and signed interaction

Sacks et al. (1974) have shown that participants in social interaction orient to a “one-at-a-time” principle for the management of turn-taking by minimizing overlapping talk between two or more participants. When overlap was observed it was rather short and regularly occurred at turn-endings. In this environment, Sacks et al. (1974) argued that the overlap manifests the next speaker's endeavor to project “*his start to be earliest possible start at some possible transition-relevance place*” (Sacks et al., 1974, p. 706f). This is particularly relevant in multi-party interaction where other participants possibly compete for the next turn. Somewhat later, Jefferson (1984); Jefferson (1986) undertook detailed analyses of the onset of overlapping turns and the timing between turns. She found that overlap onset regularly occurs within *transition space*^4^ (Schegloff et al., 1977), from which she concluded that overlap is in fact an orderly phenomenon. This does not exclude however that overlaps occur also in the midst of turns (not in transition space). However, even in the midst of turns overlapping talk is not necessarily a sign of participants' competition for the floor (French and Local, 1983). Participants can for example exploit simultaneous talk as a resource for creating interactional meaning (e.g., choral co-productions of turns for the accomplishment of agreement or the display of mutual reminiscence, Lerner, 2002). Within word search sequences, co-participants can be provided with the opportunity to overlap so as to re-establish the progression of interaction (conditional access to the turn; Schegloff, 2000). There is also possibility that overlap is actually troublesome, in which case participants repair the simultaneous talk with specific overlap resolution practices (Schegloff, 2000). In all of these cases, the model “one-at-time” is in fact warranted as a basic principle, because deviations from it are either interactionally *meaningful* or *repaired*.

For signed interaction, Cicourel (1973; cited by McIlvenny, 1995, p. 138) suggested that deaf participants may not be “constrained by the sequential ordering or chaining rules, because several signers can allow their signing to overlap continuously and several types of information can be communicated simultaneously which fall under the general notion of kinesic-visual communication.” In a similar vein, Baker (1977) stated that the visual mode of interaction in sign language “allow[s] interactants to sign and observe another's signs without a loss of understanding, whereas in oral languages, it is more difficult to hear another's speech while talking.” (Baker, 1977, p. 216). In fact, since Baker's seminal paper there have been several discussions especially on the issue of overlap in signed languages. Some scholars clearly contest the validity of Sacks et al.'s “one-at-a-time model” for sign language interactions. On the basis of informal conversations between native signers, Coates and Sutton-Spence (2001) for example observe frequent overlapping and conclude that deaf (female) signers do not orient to the interactional organization of “one speaker at a time” but to a “collaborative floor” as described in Edelsky (1981) for spoken language interaction among women. As previously Coates and Sutton-Spence (2001), also Lackner (2009) observes numerous overlaps in her data of dyadic semi-experimental conversations in Austrian Sign Language. She reports that in 3 of 13 dialogs there is a preferred “successive” structure (i.e., participants orient to the one-at-a-time organization), whereas in 4 of 13 dialogs the participants are constantly in overlap. The rest (6 dialogs) present a varying organization (Lackner, 2009, p. 94). Lackner (2009) also provides some information regarding the characteristics of the overlapping turns (cf. also Martinez, 1995). She mainly differentiates two categories. A first category is built by minimal responses such as GOOD, RIGHT, YES (for the manual minimal responses), which are often realized by smaller movements and in lower sign position than the regular signing space (i.e., they are prosodically attenuated). A second category is formed by the occurrences where the addressee initiates a short turn in overlap with the current signer for the accomplishment of a question, a complement or a comment (Martinez, 1995, p. 94). Lackner mentions that in this case the overlapped signer waits for the end of this overlap and then continues with his turn. Besides these categories of simultaneous signing, Lackner (2009) refers to two other interactional dynamics where a lot of overlaps occur. First, she mentions dialogs where participants accomplish multiple questions or comments during the telling of a participant. She observes that when a current signer is overlapped with a short question, the current signer provides an answer and continues, and the overlapping signer regularly “echoes” this answer so as to display his understanding. This echoing further results in overlap. Second, Lackner (2009) refers to moments when participants “just seem to sign simultaneously,” collaboratively constructing the dialog (complementing and referring to each other's turns). According to her, this type of interaction is related to the topic at hand (as e.g., when deafness becomes a topic of interaction). This seems similar to observations of a high-involvement signing style, as made by Coates and Sutton-Spence (2001) on American Sign Language as well as Thibeault (1993, cited by Martinez, 1995) on Filipino Sign Language interaction. All in all, several researchers working on signed interaction point out that overlaps and simultaneous signing are very frequent in signed interaction. They put forward various reasons for this, relating to contextual factors (such as the interactional topic at hand, the high involvement of signers in the interaction and shared experience), physiological factors (overlapping signals do not constrain each other) or cultural factors (women talk, sign language community).

By contrast, McIlvenny (1995) states that sequential organization is also relevant in signed interaction, and that this sequential organization is not so much affected by the fact that sign language is a spatio-visual language. More recently, several studies further pinpointed the signers' orientation to precision-timing and orientation to the coordinated transition between signers on the basis of fine-grained analyses of participants' accomplishment of turn-taking (McCleary and Leite, 2013; Groeber and Pochon-Berger, 2014; De Vos et al., 2015). For example, in a recent conversation-analytic account, McCleary and Leite (2013) provide compelling evidence for several *overlap resolution devices* (Schegloff, 2000) that deaf participants rely upon for managing overlapping signing. These more recent studies have also demonstrated the importance of a clear definition of overlap with respect to the movement phases of signs (cf. Section The Lexical Unit in Sign Language). As already pointed out by McCleary and Leite (2013), in early studies on sign language interaction it is often not clear, whether the overlap between a preparation phase and the stroke of a sign for example has been considered as an overlap or not. Indeed, considering the movement phases of signs/gestures, the following types of overlapping signing can be distinguished.

As it is the stroke or independent hold of a manual sign or gesture that houses the semantic information, an overlap between two strokes may be of a different quality than e.g., an overlap between a stroke and a hold. Indeed, as De Vos et al. (2015) have shown, signers do orient to the end of strokes as turn-boundaries (at least in question-answer sequences), and the overlaying production of retraction, preparation and hold does not seem to be troublesome (cf. also Groeber and Pochon-Berger, 2014). Nevertheless, I suggest that the other simultaneous productions may also fall under the term *overlap* as these movements are also considered as being *part of the turn* (even if they are not part of the syntactic unit; cf. Section Turn and TCU in Sign Language Research). This recalls the difference that is made in spoken interaction between overlapping conducts that are troublesome and others that are not troublesome. For example, in spoken interaction an overlap between the end of a lexical unit by speaker A with the inbreath of the incipient speaker B is of a different quality than the overlap between two lexical units (or overlaps between bodily conducts and grammatical units). An issue on this behalf is that all types of overlaps are indicated with the same transcription symbol (square brackets, [xx])—as mentioned in Groeber and Pochon-Berger (2014), the use of various transcription symbols for different types of overlaps may become relevant for documenting such differences, both in signed and in spoken interaction.

## Method

### Participants and data

The current analysis is based on a 33-min four-party interaction in Swiss German Sign Language (*Deutschschweizerische Gebärdensprache*, henceforth: DSGS) between four young deaf women: Denja, Nathalie, Isaline and Melinda. All participants have deaf and signing parents and learned DSGS as their L1. The data comes from a larger corpus of DSGS narratives and interactions that has been gathered within the project “*Gaze and Productive Signing in a Corpus of Interactions of Deaf and Hard-of-Hearing Signers of Swiss German Sign Language (DSGS)*,” conducted at the *University of Applied Sciences of Special Needs Education*, and funded by Swiss National Science Foundation. Within this project, participants were invited to attend to a whole day of recording at a film studio producing programs in sign language. While there are some semi-experimental data, the film under study here comes from the lunch break that was recorded with the aim of having a maximally natural interaction. All data was filmed with three cameras. Two cameras were positioned to record two participants frontally, and the third camera captured all participants together.

### Procedure and analysis

#### Annotation

The annotation was done in *iLex*, a corpus-annotation tool developed at University of Hamburg for sign language documentation (e.g., Hanke, 2002), which the project team uses for the previously cited project. The annotation was then exported in *eaf*-format for an import into the multimedia annotation tool ELAN^5^. For the analysis, several tiers have been added to the ELAN-annotation (cf. Section Analysis).

In the current state, the basic transcript consists of a content translation (done by two interpreters), an annotation of manual signs and participants' gaze conduct. Manual signs have been annotated with *glosses*, i.e., words taken from the spoken language (in our case German spoken language) that roughly describe the meaning of a sign. These glosses function as labels for a sign and are not a precise translation. In the transcript they are always written in capitals. As the project team is working with *iLex*, the glosses in the transcripts are automatically linked to the sign databank for Swiss German Sign Language. When a sign is not available in the lexicon, the annotators create a new gloss, which is automatically added to the lexicon.

In this project we segmented lexical signs in a broad way, i.e., the sign starts with the preparation or the transition phase of the sign, and it ends with the end of the stroke. This implies that the end of sign 1 *is* the beginning of sign 2, and that there are no gaps between signs (cf. Figure 1, Section The Lexical Unit in Sign Language; Hanke et al., 2012 on sign segmentation). The annotation by the gloss therefore includes the preparation phase or the transition phase of the sign as well as the stroke of the sign. The stroke starts with the full deployment of the sign's handshape and initial orientation and position. The stroke ends when all manual parameters of the sign are realized (handshape, orientation, location, and movement) (cf. also De Vos et al., 2015). By contrast to De Vos et al. (2015) and McCleary and Leite (2013) we did not include the retraction phase, the return to rest position, into the gloss. For the excerpts under discussion in this article, we added a notation of the gesture phases (Kita et al., 1998) of the manual signs, making the types of overlapping signing more explicit (McCleary and Leite, 2013; De Vos et al., 2015). The detailed transcription conventions are represented in **Table 2**.

A range of other non-manual components such as eyebrows or mouthings^6^ are important aspects of sign language. They will be regarded as such in the analysis, but they are not (yet) systematically annotated over the whole corpus.

#### Analysis

In what follows I briefly outline the different analytical steps that I undertook for the current study once the glosses and gaze conducts have been annotated in ELAN.

**Figure 3:**
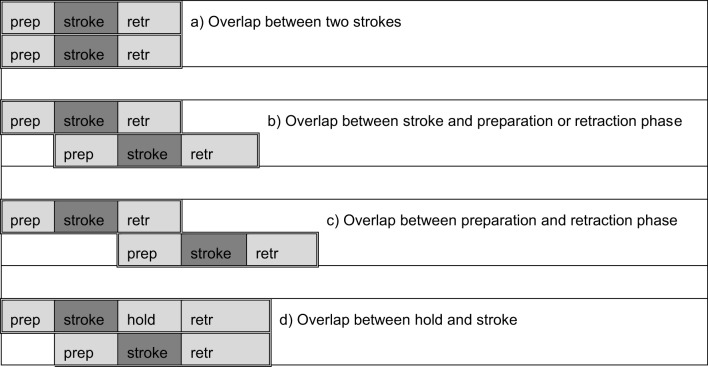
**Different types of overlaps**.

(1) *Identification of those overlaps relevant for the current study*. As mentioned in Section The Turn-Taking Machinery in Signed Languages, there are different types of overlaps with respect to their quality. In this study the analysis is limited to one specific type of overlap, by excluding:
  - When the preparation phase of a participant's sign overlaps with the stroke or the retraction phase of another participant's sign; the reason is that there is no overlap on a semantic level (cf. Figure 3, cases b and c in Section Overlaps in Spoken and Signed Interaction; cf. De Vos et al., 2015).
  - When a participant is holding a turn-final sign for a moment and another participant produces a sign that is overlapping the hold (cf. Figure 3, case d, in Section Overlaps in Spoken and Signed Interaction, cf. Groeber and Pochon-Berger, 2014). The reason is that the end of the stroke may constitute a first TRP, while the hold is extending the turn. There is no overlap on a semantic level.
  - When non-manual conducts such as head shake or head nod from one participant overlap the manual sign of another participant. This decision is due to the fact that I did not look at the non-manual turns; whether such types of overlaps should be considered as overlaps to the same extent as overlaps between signs is a question that cannot be addressed in this paper.

Each overlap was tagged in ELAN on a separate analysis tier. On the basis of participants' gaze conduct I then chose to distinguish between two types of overlaps. Most frequently, overlaps occur between participants who are gazing at each other, either at the beginning, the end or over all their turns. This implies that at some point there is mutual orientation and participants can perceive their simultaneous production. Another type of overlap consists of simultaneous signing between two participants while they are addressing a third person through their action and gaze conduct. This type of overlap can go totally unnoticed by the participants (i.e., there is no mutual orientation; cf. also McIlvenny, 1995 on that topic of simultaneous turn beginnings). Such overlaps occur frequently after lapses. The data under study presents a total of 382 overlaps. Out of these, 331 instances occur between two or more participants who are gazing at each other at some point during the overlap. In the remainder of this paper, only those 331 cases are further investigated.

(2) *Description of the overlapping turn with respect to action*. For each overlap I attempted to tag the action that the participants accomplished with that turn. While some turns were easily interpretable on the basis of the *next-turn proof procedure*^7^ (Sacks et al., 1974, p. 728), other actions were more difficult to determine, especially in those courses of action that consist of storytellings.
(3)*Description of the sequential environment of the overlapping turn's onset with respect to the overlapped turn*. The analysis of all relevant overlaps resulted in a categorization of the overlaps in three main sequential environments:
  - The overlap occurs at a first possible completion place reached by participant A (cf. cases A and B in the table below).
  - The overlap occurs near the end of a unit or the potential end of a unit (but not exactly at the transition place) (cf. cases C and D in the table below).
  - The overlap occurs in the midst of a unit (cf. case E).

## Sequential environments of overlapping signing

This section provides an overview of different types of simultaneous signing that can be observed in the data under study. While several authors have commented on the types of actions that are implemented by overlapping turns (e.g., minimal response, repair, short queries, or comments; Martinez, 1995; Coates and Sutton-Spence, 2001; Lackner, 2009), the sequential environments of the overlap onsets have not yet been differentiated systematically in previous studies on signed languages. For this differentiation, I investigate the sequential environments where an incipient signer overlaps an ongoing turn of a current signer. It is important to highlight that the analytic focus is primarily on the sequential environment at the *turn-level*, i.e., whether the overlap occurs at the beginning, in the midst or the end of a possible turn. The overlap onset at the lexical level (i.e., with respect to the movement phases within isolated signs) is visible also in the transcripts, but it is not the focus of analysis in this paper (but see De Vos et al., 2015).

### Simultaneous signing at places of possible completion

**Figure 4:**
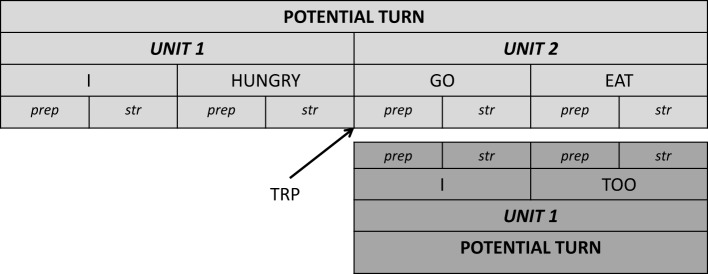
**Overlap onset at first possible completion**.

One turn environment where simultaneous signing occurs is at first possible completion of a signer's unit (Table 1, cases A and B; cf. also Jefferson, 1984). At the precise moment for example where participant A (light gray in Figure 4) reaches a possible end of a turn, e.g., the end of the interrogative unit *I HUNGRY* (marked by the end of the stroke of the sign *HUNGRY*), another participant B (gray in Figure 4) may launch into a turn. If participant A actually does not continue after this completion, we would observe a smooth turn transition. If however, participant A continues after that first possible completion while participant B also launches into a turn, an overlap occurs^8^.

**Table 1:** **Categories of overlap onset tagged in ELAN**.

	**Category**	**Overlap onset with respect to the current signer's turn**	**Explanation (Participant A = current signer; participant B = incipient/overlapping signer)**	**Quantification**
A	Start of new unit	At a first TRP, i.e., after the stroke of the potentially last sign	Both participant A and participant B launch a unit which is syntactically independent from what precedes (new syntactic construction); this can occur after a pause or straightforwardly after a TRP	110/331 (33.2%)
B	Extension of unit	At a first TRP, i.e., after the stroke of the potentially last sign	Participant B launches a turn and participant A adds one or several signs after his initial TRP; these signs are dependent from the first part of the turn; this can occur after a pause or straightforwardly after a TRP	
C	End of unit	During the stroke of the last item of a turn	Participant A produces the last item of his turn (and then retracts his hands), participant B's turn-initial sign (stroke) overlaps the deployment phase (stroke) of that last item	7/331 (2.1%)
D	Potential end of turn followed by continuation	During the stroke of an item that *could* be the last item of the turn	Participant A produces an item that could be the last item of the turn, participant B's turn-initial sign (stroke) overlaps the deployment phase (stroke) of that potentially last item—but after that item A continues with additional signs or with a new (syntactically independent) unit	146/331 (44.1%)
E	Midst of unit	After the beginning of a syntactic unit, not in potential transition space	Participant B launches a turn while participant A's turn is not yet reaching a possible completion	35/331 (10.6%)
F	Undetermined			33 (10%)
Total overlaps				331

An additional aspect to the overlap onset pertains to the design of overlapped turns. When participant A continues with his turn after a possible completion, this continuation can be independent from the first part (Section Overlapping a Current Signer's New Unit at Places of Possible Completion), or it can be syntactically dependent (Section Overlapping a Current Signer's Dependent Unit at Places of Possible Completion).

#### Overlapping a current signer's new unit at places of possible completion

The first excerpt illustrates an overlap occurring at a place of possible completion between a current signer, Nathalie, and a new signer, Melinda. The segment stems from a repair sequence initiated by Melinda concerning an exchange that she missed between Nathalie and Isaline. Isaline said that she really dislikes her eyes without make-up because they appear so big. Nathalie responds to this by expressing her surprise. During this exchange, Melinda averts her gaze from the participants while she is drinking (she presumably sees however Isaline saying that she dislikes something and the expression of surprise by Nathalie). After Nathalie's display of surprise, Melinda initiates repair by asking Nathalie what Isaline dislikes. When Nathalie reaches a potential completion of her repair, Melinda starts a turn (addressed to Isaline) while Nathalie is continuing with a “new” unit, resulting in simultaneous signing.

**Figure 5:**
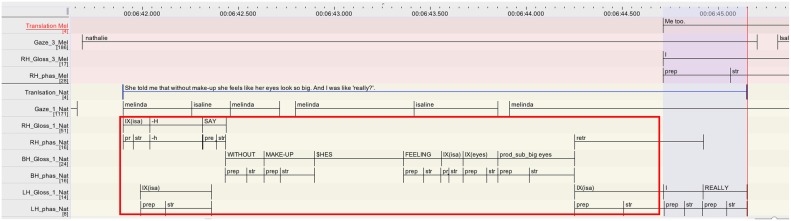
**Annotation grid excerpt 1, corresponding to lines 07–09**.

The excerpt is presented as follows: a rough gloss annotation of the larger sequence is provided first, followed by a content translation. The part of these transcripts that are in bold are then represented again in the format of the ELAN annotation, so as to provide some more details on the temporal unfolding of the overlap under discussion (Figure 5). In the ELAN annotation, each participant is represented with a different color and comprises several tiers. The labeling of the tiers is further explained in the transcription conventions (Table 2).

**Table:** **Excerpt 1** (Corpus InterGaze, 00:06:41)

**Gloss annotation of larger sequence**		
01	Isa:	I HATE I NOT MAKE-UP prod-sub-eyes
02		HATE I
03	Nat:	REALLY YOU REALLY
04	Mel:	[WHAT HATE WHAT
05	Nat:	[I LIKE prod-skizz-small eyes LIKE TIRED
06		(FALL-ASLEEP DEAD) I prod-skizz-small eyes WITHOUT
**07**		**IX(isa) WITHOUT MAKE-UP $HES IX(eyes) prod-sub-big eyes IX(isa)**
**08**		**[I REALLY**
**09**	**Mel:**	**[I TOO** I
**Translation of larger sequence**		
01	Isa:	I hate my eyes without make-up – they look so big,
02		I hate that
03	Nat:	Oh really?
04	Mel:	[what does she hate?
05	Nat:	when I don't put make-up my eyes appear very small
06		I look like exhausted without make-up
**07**		**she told me that without make-up her eyes look so big**
**08**		**[and I was like ‘really?’**
**09**	**Mel:**	**[me too**

**Table 2:** **Annotation conventions**.

**TIERS IN ELAN (PER PARTICIPANT)**	
Translation_Nat	Tier for content translation of Nathalie's contribution
Gaze_1_Nat	Gaze conduct of Nathalie (participant 1)
RH_Gloss_1_Nat	Gloss for the sign produced on right hand by Nathalie
LH_Gloss_1_Nat	Gloss for the sign produced on left hand by Nathalie
BH_Gloss_1_Nat	Gloss for the sign produced on both hands by Nathalie (for two-handed signs)
RH_phas_Nat	Gesture phases for the sign produced on right hand
LH_phas_Nat	Gesture phases for the sign produced on left hand
BH_phas_Nat	Gesture phases for the sign produced on both hands
**TRANSCRIPTION CONVENTIONS FOR THE HAND TIERS RH_GLOSS_1_NATH ETC**.	
BALL	Gloss for standardized sign of DSGS
BALL-pl	Plural for BALL
IX(isa)	Pointing toward the person/object in brackets
B-A-L-L	Fingerspelling/Fingerspelled letters (B, A, L)
PALM-UP	A sign/gesture with palms oriented upwards
PALM-DOWN	A sign/gesture with palms oriented downwards
$HES	Hesitation or interrupted sign
D-HAND	Hand configuration of a fingerspelled D, but does not have a clear directional movement as a pointing
prod-man-needle	Productive sign (semi-lexical sign) that consists of a *depiction* of an object, animate referent or a situation (cf. also depicting signs). Productive signs are annotated with the *image producing technique* they use (Langer, 2005) and with a reformulation of what they represent Image producing techniques: **Man**: manipulative technique (cf. also handle classifier) **Skizz**: sketching technique (cf. also size and shape specifier) **Sub**: substitutive technique (cf. also entity classifier)
-H	Hold of a sign/gesture
**TRANSCRIPTION CONVENTIONS FOR THE MOVEMENT PHASE TIER**	
prep	Preparation of the sign/gesture, i.e., movement out of rest position
str	Stroke
retr	Retraction
-H	Hold
**TRANSCRIPTION CONVENTIONS FOR THE GAZE TIER**	
D, DD	To the right
G, GG	To the left
D: down	Down
den, nat, isa, mel	Toward Denise, Nathalie, Isaline or Melinda

Nathalie's repair turn (l.07-08) consists of two parts. She starts with reporting that Isaline told her that without make-up Isaline's eyes look so big (red rectangle). This part finishes with the referential pointing toward Isaline (*IX(isa)* on the tier LH_Gloss_1_Nat; l.07) which is doubling the same pointing at the beginning of the unit. This type of reduplication of a pronoun is frequent in several signed languages and has been described as a prosodic marker (e.g., Crasborn et al., 2012). Nathalie then launches into the second unit of her turn with *I REALLY “I said ‘really?’* (on the tier LH_Gloss_1_Nat; l.08), reporting her response to Isaline (highlighted in blue).

It is in overlap with this second part of the turn, that the repair initiating party Melinda takes a turn that displays her re-established understanding by affirming that she has the same problem (*I TOO I*, “me too,” on the tier RH_Gloss_3_Mel; l.09). Interestingly, Melinda's response to Nathalie's repair is finely tuned to the moment when the element she addressed as repairable has been mentioned, namely *what* does Isaline dislike (“her big eyes”). At this point, she therefore orients to pragmatic completion, as her repair initiation did not ask for anything more than “what does she/Isaline hate.” Note also that Melinda launches a response to the repair after the reduplication of the sign *IX(isa)*, where the first part is markedly finished on a syntactic and prosodic level. What we see here is thus precisely what Jefferson (1986) describes as possible completion onset, where “*[a] recipient reasonably, warrantedly treats some current utterance as complete, ‘transition ready,’ and starts to talk, while (..) the current speaker, perfectly within his rights, keeps going*.” (Jefferson, 1986, p. 154). Considering the movement phases of the overlapping signs, it is interesting to note the following: while Nathalie and Melinda overlap each other by respectively extending and launching a turn, it is only the stroke of *REALLY* (LH_phas_Nat) that is overlapped with a stroke by Melinda (RH_phas_Mel). The other overlaps concern strokes and preparation phases. Interestingly, the preparation phase of Melinda's *I* (RH_phas_Mel) is rather long (350 ms)^9^ —it may be possible that Melinda stretches the preparation phase of *I* in response to the fact that Nathalie is extending her turn (overlap resolution device; McCleary and Leite, 2013).

The excerpt illustrates a regular way of turn transition between signers: incipient signers do not necessarily wait for the current signer relinquishing the floor by retracting the hands to rest position. Rather they fine-tune their turn-beginnings to the end of grammatical and prosodic units (cf. also Selting, 1996, 2000 for spoken interaction) marked by the stroke of the turn-final sign. This is similar to the phenomenon observed in spoken interaction, where participants do not normally wait for a pause after a turn for launching a new turn. By contrast, participants finely monitor ongoing turns for their actional, syntactic and prosodic completion^10^. In the present data 33.2% of all overlaps occur at potential completion points where participants reasonably guess that a turn is finished (cf. Table 1), while the current participant continues past possible completion.

In excerpt 1, the current signer continues the turn with a second unit that is syntactically independent from what precedes. By contrast, excerpt 2 illustrates a case where the signer's continuation consists of a unit that is dependent on the initial unit.

#### Overlapping a current signer's dependent unit at places of possible completion

This type of overlap occurs when participant A reaches potential completion, and participant B launches a turn while participant A continues with one or several further signs that are dependent from the first part. The sequential onset of overlap by the incipient signer is therefore exactly the same as described in Section Overlapping a Current Signer's New Unit at Places of Possible Completion, but the design of the overlapped turn part of the current signer is different.

**Figure 6:**
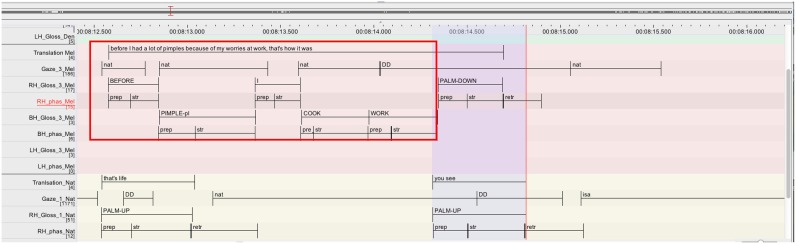
**Annotation grid excerpt 2, corresponding to lines 04–06**.

Excerpt 2 presents a simple case of a short overlap between Melinda and Nathalie within a discussion on the different possible origins of pimples (Figure 6). Melinda tells that in the past she had a lot of pimples because of her worries at work (l.04). After this first unit she extends her turn with a second unit, a *PALM-DOWN* gesture, which could be translated as “that's how it was” (l.05). This second part of the turn is overlapped with Nathalie's acknowledging response that can be translated as “you see” with a *PALM-UP* gesture directed toward Melinda (l.06).

**Table:** **Excerpt 2** (Corpus InterGaze, 00:08:12)

**Gloss annotation of larger sequenc**		
01	Nat:	I MAINLY I STRESS I [I D-HAND
02	Mel:	[I TOO I
03	Nat:	[PALM-UP
**04**	**Mel:**	**[FORMER PIMPLE_pl I COOK WORK**
**05**		**[PALM-DOWN**
**06**	**Nat:**	**[PALM-UP(mel)**
**Translation of larger sequence**		
01	Nat:	I have them mainly due to stress
02	Mel:	me too
03	Nat:	[that's life
**04**	**Mel:**	**[before I had a lot of pimples because of my worries at work**,
**05**		**[that's how it was**
**06**	**Nat:**	**[you see**

Nathalie launches her acknowledging response to Melinda's telling, a *PALM-UP* gesture directed to Melinda (RH_Gloss_1_Nat; l.06), in precise overlap with the last item of Melinda's turn, the gesture *PALM-DOWN* (RH_Gloss_3_Mel; l.05). Considering the movement phases of the overlapping gestures, it becomes clear that Nathalie launches her responsive *PALM-UP* near the end of the stroke of Melinda's sign *WORK*, the final sign of her first unit. Thus, Nathalie orients to this moment as reaching potential completion where she can acknowledge Melinda's telling and display her understanding. Melinda's turn however continues with a sign *PALM-DOWN*, which she adds without any manual prosodic disruption (i.e., no pause or slowing down before *PALM-DOWN*). Despite the absence of a manual prosodic break after *WORK*, Melinda deploys a resource that indicates a possible completion after *WORK*—she averts her gaze from her co-participant (to the right side) during the deployment of *WORK* (cf. DD on the tier Gaze_3_Mel). Baker (1977) described gaze aversion from co-participants as recurrent in turn-*beginnings*, where they are exploited as a resource for displaying cognitive planning and holding the turn (cf. also Kendon, 1967). Turn-endings have by contrast have been described as being accompanied with a gaze oriented to the co-participants, indicating thereby that the current participant yields the turn to a next signer (Martinez, 1995; Baker, 1977). This has been observed also as predominant in question-answer sequences in a dyadic teacher-student interaction in Swiss German Sign Language (Groeber, 2011). Both the teacher and the student orient their gaze toward their co-participant at the end of both questions and answers (in 90–100% of cases). An important difference with the excerpt under study here is however that after Melinda's telling (l.04) no projection for a next action is pending (Auer, 2002). The topical talk can continue (as it actually will by Isaline taking a turn), or it could also be closed down. This clearly contrasts with the collection under study in Groeber (2011), where the questions set a strong projection for information provision, while the answers set a projection for an evaluation (cf. three-turn structure in teaching context; Mehan, 1979). In the light of these observations, Melinda's gaze aversion during *WORK* rather indicates the upcoming completion of a sequence similarly to what Rossano (2012) describes for spoken language. Consequently, the addition of *PALM-DOWN* at the end of Melinda's turn can be qualified as a resource that enables Melinda to smoothly step out of her turn. This extension by means of a short non-lexicalised item results in an overlap that is similar to the turn-final overlaps of tag questions described by Selting (1996; cf. Section Turn and TCU in Classic CA). In fact, the excerpt represents a recurrent way of how signers in the present data end their turns. We will take this issue up again with excerpts 3 and 5.

Excerpts 1 and 2 have shown the fine coordination of incipient signers to possible completion, which implies that the overlapping signing is not the result of mistiming. Signers therefore precisely time their turn beginnings to such places within the temporal unfolding of turns, where syntactic boundaries occur. The overlaps are the result of the fact that the current speaker is not ending her turn at this point, but extends her turn.

### Simultaneous signing after places of possible completion

**Figure 7:**
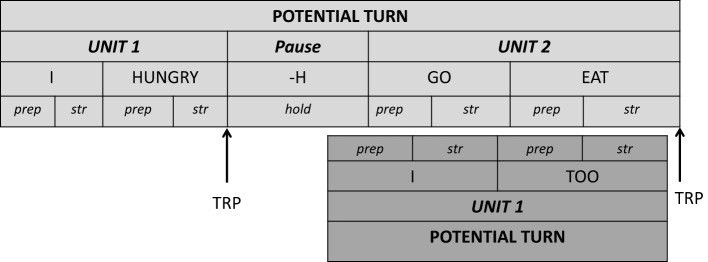
**Overlap onset after first possible completion**.

A further environment where simultaneous signing occurs is just after a possible completion of a signer's unit, i.e., when some pause has occurred after the first possible completion place (i.e., the end of the stroke of the turn-final sign). This pause can consist of a full or partial retraction, or it can be filled with a hold^11^. The case is schematized in Figure 7 below.

**Figure 8:**
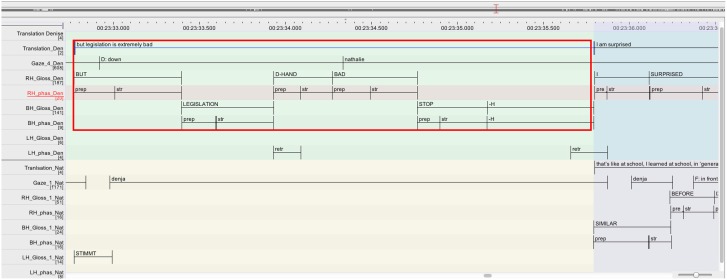
**Annotation grid excerpt 3, corresponding to lines 02–04**.

When participant A continues with his turn after some pause after a possible completion, he can do this either by starting a new unit or by adding elements that are somehow dependent on the first part of the turn (as in Section Simultaneous Signing at Places of Possible Completion). In this section I present an example of the first case in excerpt 3 (Figure 8). Denja finishes a first unit (l.02), followed by a pause (0.6 s with a hold). After this pause she launches a second unit (l.03) at the same time as her co-participant Nathalie (l.04).

**Table:** **Excerpt 3** (Corpus InterGaze, 00:23.32)

***Gloss annotation of the larger sequence:***		
01	Den:	SECOND SECURITY IMPORTANT GOOD
**02**		**BUT LEGISLATION D-HAND BAD STOP +(0.6) −H+**
**03**		**[I SURPRISED I +(0.4) −H+]**
**04**	**Nat:**	**[SIMILAR BEFORE IX(v) SCHOOL I]**
		SCHOOL I LEARN –A-B-U-^12^ I TOO I TOPIC LEGISLATION…
***Translation of the larger sequence:***		
01	Den:	security is important and very good
**02**		**but legislation is bad**
**03**		**[I am surprised**
**04**	**Nat:**	**[that's like at school**
**05**		**I learned at school**, in ‘general knowledge’ we also treated the topic legislation ….

The segment starts with Denja affirming that Switzerland is a very safe country, but that there is poor legislation resulting in too mild sentences, as for example for the punishment of rapists or murderers (*BUT LEGISLATION D-HAND BAD STOP -H*, on the tiers RH_Gloss_Den/BH_Gloss_Den; l.01-02, with headshake that starts in parallel to the sign *BAD*). At the end of the sign *BAD*, Denja's turn reaches a first possible syntactic and pragmatic completion. She continues however with the sign *STOP*, after which again speaker change may occur. This sign *STOP* is then held for 0.6 s, and she expands her headshake that she began in parallel to the sign *BAD*). Nathalie provides an acknowledging response by means of a head nod that is precisely timed to the end of the sign *BAD*; this head nod extends simultaneously to the sign *STOP* and the further *-H (hold)*. With this non-manual acknowledgment the course of action has reached some completeness and there is no constraint regarding who of the participants takes a turn. Nathalie might take a full turn during Denja's *-H*, i.e., she could launch a turn going beyond her non-manual acknowledgment, but her hands remain in rest position. By contrast to Nathalie's turn-ending in excerpt 2, where her gaze aversion from the co-participant was interpreted as a possible sequence closing resource, Denja is gazing at Nathalie during her *-H*. I suggest that by means of these resources she invites Nathalie to take a turn and elaborate on the topic (cf. Stivers and Rossano, 2010 for spoken language). In a recent contribution, Groeber and Pochon-Berger (2014) proposed that turn-final holds embody the participant's expectation of the fulfillment of a pending action (as e.g., an answer to a question). In the current excerpt, the turn-final hold also contributes to the embodiment of an expectation for a continuation, even if there is no action projection under way, i.e., there are no constraints (in terms of sequence organization) on how the interaction is expected to continue. Both Nathalie's turn-launching at the end of Denja's hold and Denja's further simultaneous turn extension corroborate this idea. Denja finally continues her turn by adding to her first part of the turn (red rectangle) that this is what she realized (*I SURPRISED I*, tier RH_Gloss_Den; l.03). In overlap with the preparation phase of *I*, also Nathalie finally takes a turn by launching the preparation phase of her turn-initial sign *SIMILAR* (tier BH_Gloss_1_Nat; l.04), orienting thereby to Denja's previously deployed resources (*-H and gaze*) as yielding the floor to her. This simultaneous beginning after the completion point results in overlap (highlighted in blue).

I suggest that from the end of *BAD*, Denja creates a negotiation space (over the signs *STOP* and its *-H*) where participants deal with the determination of a next signer in a subtle and situated manner. This is a most relevant interactional task especially in moments where no projection is pending, which means that there is neither constraint with respect to the next action to be accomplished, nor with respect to whom of the participants will get the floor (cf. also excerpt 2).

It is interesting to note that when Nathalie has finally taken over the turn, Denja does not drop out of the overlap immediately, but she brings her new unit to an end (l.03). However, Nathalie clearly orients to the simultaneous signing as a potentially troublesome overlap, as she restarts the overlapped turn-beginning (*SCHOOL I*, l.04) as soon as the simultaneous signing quits.

In Sections Simultaneous Signing at Places of Possible Completion and Simultaneous Signing after Places of Possible Completion, I have shown that overlaps in signed interaction are, in a lot of cases, orderly (Jefferson, 1984, 1986). They can be a result of the participants' orientation to syntactic and pragmatic completion points of current signers, by launching a turn either at the first possible completion point (stroke of turn-final sign), or slightly past a first possible completion point (after a short pause). In what follows I show that participants can also anticipate an upcoming completion point and launch a turn while the current signer is approaching a first possible completion point (cf. Table 1, categories C and D; 153/331 overlaps, 46.2%).

### Simultaneous signing before places of possible completion

**Figure 9:**
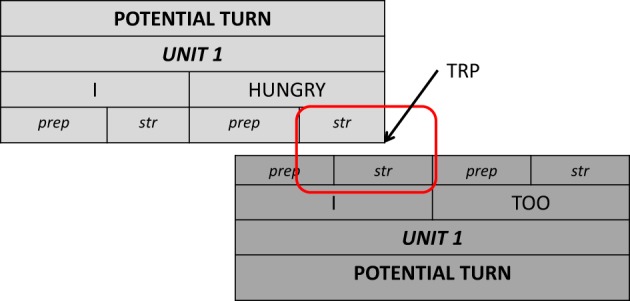
**Overlap onset before first possible completion**.

The anticipation of an upcoming possible completion can result in different types overlap. Relevant for this study are those overlaps where the stroke of participant A's final sign overlaps with the stroke of participant B's final sign. This is illustrated in Figure 9 below.

The anticipation can be correct, and the participant A finishes his turn within one sign (as in Figure 9 and Section Overlapping a Current Signer's Last Item of a Unit), or it can be erroneous and the participant A continues, either with more than one sign, or even with a new syntactic unit (Section Overlapping a Current Signer's Potentially Last Item of a Unit).

#### Overlapping a current signer's last item of a unit

**Figure 10:**
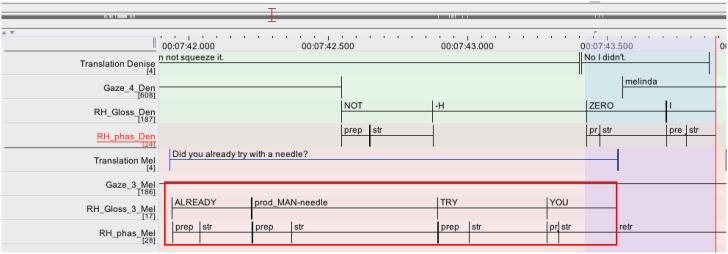
**Annotation grid excerpt 4, corresponding to lines 03–05**.

In this section I present an illustration of such cases where a participant B starts signing while participant A is producing the last sign of a unit. These types of overlaps show that participants can foreshadow potential turn-endings. This corresponds to what Jefferson (1986) treats as *terminal onset* and more precisely “*last item*” *onset*, where the final sounds of a last word by a current speaker are overlapped with the beginning of a new speaker. In excerpt 4, Denja and Melinda are talking about Denja's pimple which she has had for 2 weeks (Figure 10).

**Table:** **Excerpt 4** (Corpus InterGaze, 00:07:41)

***Gloss annotation of the larger sequence:***		
01	Den:	(XX) THEREUNDER SEE YELLOW THERE IX(pimple) PALM-UP
02		[prod-man-squeeze pimple CAN NOT -H
**03**	**Nat**	**[(YOU) ALREADY prod-man-needle TRY [YOU**
**04**	**Isa:**	**[$HES WAVE(isa)**
**05**	**Den:**	**[ZERO I**
***Translation of the larger sequence:***		
01	Den:	(xx) one sees it's yellow
02		but I cannot squeeze it
**03**	**Mel:**	**[did you try with a needle [(you)?**
**04**	**Isa:**	**[euhm denja**
**05**	**Den:**	**[no I didn't**

Denja is reporting that she was not able to squeeze the pimple despite the fact that it was all yellow. In overlap with this (which is not the focus of this analysis), Melinda is addressing a question to Denja, namely whether she has already tried to pick it with a needle [*(YOU) ALREADY prod-MAN-needle TRY YOU*, tier RH_Gloss_3_Mel; l.03; red rectangle]. Denja launches an answer (*ZERO I*, “no I didn't,” tier RH_Gloss_Den; l.05) to this question while Melinda is still producing the stroke of her last sign of her question, the sign *YOU* (l.03; highlighted in blue). Such examples show that signers can project incipient turn completions and launch into the transition even before the current signer has actually finished. Surprisingly, in the data under study here at least, cases where a signer provides minimal responses or launches a new turn in overlap with a sign “under way” (during the stroke deployment) that will actually be the last sign of the turn are very rare (7/331 overlaps; 2.1%; cf. Table 1). For those minimal responses and turn starts that arise in overlap, it is much more frequent that they occur during the deployment of a *potentially* last item, i.e., that after that item the current signer continues with her turn (cf. also Section Simultaneous Signing at Places of Possible Completion). In the next section I will focus on those instances in more detail and propose a discussion on the possible reasons for the frequent accomplishment of this type of turn transition.

#### Overlapping a current signer's potentially last item of a unit

**Figure 11:**
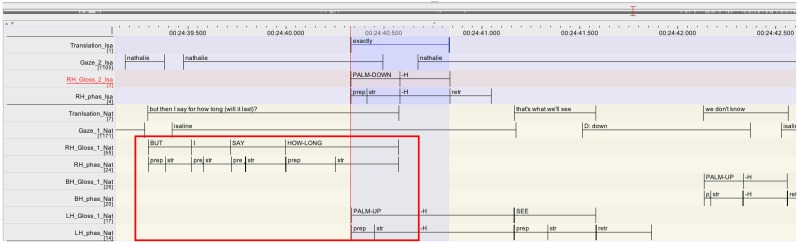
**Annotation grid excerpt 5, corresponding to lines 02–06**.

Current signers are recurrently overlapped during a potentially last item of a unit, after which they continue their turn (146/331; 44.1%; cf. Table 1). Frequently the overlapping participants are providing only short acknowledgments, hence they are not claiming the floor and the current signer can continue without any disruption. While such turn continuations may have been projected (as e.g., in a storytelling before the climax), other continuations rather seem to occur in the absence of a fuller turn taking by a potential next speaker. Excerpt 5 presents such a case (Figure 11). In excerpt 5, Isaline's acknowledging response (l.04) is overlapping the potential end of Nathalie's turn (l.03). The two women are talking about rapists and appropriate prison sentence.

**Table:** **Excerpt 5** (Corpus InterGaze, 00:24:32)

***Gloss annotation***		
01	Nat:	EXAMPLE (NAME) PALM-UP
02		GOOD PALM-UP
**03**		**IX(name) BUT I SAY (xx) HOW[-LONG**
**04**	**Isa:**	**[PALM-DOWN**
**05**	**Nat:**	**WE-SEE**
**06**		**PALM-UP**
***Translation of larger sequence:***		
01	Nat:	for example (name)
02		(okay)
**03**		**(name) but then I say for how long (will it last)?**
**04**	**Isa:**	**yes exactly**
**05**	**Nat:**	**that's what we'll see**
**06**		**we don't know**

Nathalie says that she wonders for how long a rapist who is freed from prison will go before relapse (*BUT I SAY HOW-LONG*, ON TIER RH_GLOSS_1_Nat; l.03). The sign *HOW-LONG* (produced on the right hand) is accompanied with a head tilt back and raised brows (question design; Boyes Braem, 1990). Isaline orients to this moment in Nathalie's turn as potentially reaching some completion; in overlap with the stroke of *HOW-LONG* (l.03) she deploys an acknowledging response that consists of a lifting of a *PALM-DOWN* gesture with a prolonged mouthing of the letter—e-^13^ (this could be translated as “exactly” or “yes that's it”). Simultaneously to the preparation phase of Isaline's acknowledgment *PALM-DOWN*, also Nathalie lifts her left hand (while on the right hand she still signs *HOW-LONG*) and produces a *PALM-UP* (tier LH_Gloss_1_Nat; l. 06), which she holds for a moment.

Interestingly, when Nathalie sees Isaline's preparation phase of her acknowledgment (raising the hands out of rest position), Nathalie could relinquish the floor by retracting her hands into rest position (i.e., after the sign *HOW-LONG*). However, Nathalie still prepares and holds her *PALM-UP* on the left hand until Isaline has again retracted her hands to rest position. Different hypotheses can be drawn up for explaining Nathalie's *-H*: first, Nathalie may thereby indicate that she does not want to relinquish the turn, i.e., that she is holding the floor. However, *-H* as a floor-holding device has been described as being accompanied with gaze aversion from the participants (Baker, 1977). Moreover, as the continuation of the excerpt shows, Nathalie will not add a lot of material after this *-H* but rather launch into a closing of the sequence (l.05-06). A second possibility is that, by means of her *-H*, Nathalie embodies her expectation of Isaline's response (Groeber and Pochon-Berger, 2014). However, when Nathalie starts holding her sign, Isaline has already provided a response with her acknowledgment. There is thus not strong evidence for this explanation. A possible explanation that was already mentioned for excerpts 2 and 3, is that Nathalie uses both the *PALM-UP* and the *-H* as resources for a smooth stepping out of the turn, creating thereby the opportunity for a turn transition that is not abrupt but fluid. While Isaline is invited to take a turn beyond her acknowledgment, Nathalie does not immediately end her turn but fills in the delicate moment of transition with these resources. This is further corroborated with Nathalie's continuation in the absence of Isaline's turn-taking. Nathalie extends this negotiation space with the extension by *SEE* (“that's what we'll see,” tier LH_Gloss_1_Nat; l.05) during which Nathalie averts her gaze from the co-participant (cf. excerpt 2 for a similar case). It is only after the simultaneous and sequential combination of all these resources that Nathalie retracts her hands to rest position. However, even after having retracted her hands to rest position (on the table), she then further extends the turn with a short *PALM-UP* accompanied with a shrug of the shoulders (“we don't know,” tier LH_Gloss_1_Nat; l.06). This is again accompanied with a glance toward Isaline, who still does not take a turn.

In sum, this excerpt showed that Isaline overlaps a possible last item of Nathalie's turn. Nathalie continues with some further signs, which seem to be deployed as a resource for the smooth management of turn transition or turn endings. It is interesting to note that this subtle stepping out of a turn is again occurring at a moment where no specific action projection is set for the continuation of the sequence (cf. excerpt 2).

Up to this point I focused on those overlaps that occur within transition space, corroborating the orderliness of overlapping signing as it has been shown in spoken interaction. A further sequential moment where overlaps occur is what I term *within units*. Jefferson (1986) refers to such overlaps has having an “interjacent onset.”

### Simultaneous signing within units

A last sequential moment of overlapping signing is when participant B launches a turn in the midst of a syntactic construction by participant A. This type of overlap is rare in the data under study (35/331 overlaps, 10.6%). While the sequential moment of these overlaps does not manifest any coordination between the participants with respect to turn management, an interesting question to ask is what actions participants accomplish by means of these overlapping turns. In what follows I provide a brief description of the principal actional environments in which these overlaps occurred. Note however that each case is very specific with respect to the involvement of the participants and the coordination processes (mutual gaze and orientation).

A first environment where participants overlap each other with interjacent onset is in courses of actions involving repair (7/35 cases). The overlapping party is either self-initiating other-repair, providing a self-initiated other-repair, or displaying his understanding after having launched a repair sequence (sequence-closing third; change-of-state token, Heritage, 1984). As the establishment and maintenance of intersubjectivity is a condition for social interaction to happen, repair appears to be an action that is legitimately accomplished at any place. A second environment of interjacent overlap is built by those moments where participants either display their early understanding (by reformulating a current signer's turn) or by displaying that they share experience with the current signer. By these overlapping turns participants exhibit their alignment with a participant, and at the same time they inform each other about their epistemic status with respect to what is being told (10/35 cases). A third environment is built by courses of action where the overlapping participants display their strong disagreement with a current signer's turn (3/35).

Sometimes participants also provide acknowledgments that are not fine-tuned to transition space but come in midst of a syntactic construction. In 3 of 4 cases, this acknowledgment ensues the introduction of a reference by means of list construction.

## Discussion

### The orderliness of overlaps in multi-party signed interaction

The turn-taking organization is one of the most basic analytic tools we have in CA for the analysis of broader interactional phenomena (such as repair organization or preference). In order to understand how members of a community accomplish courses of action, and establish and negotiate meaning in a situated and dynamic way, we have to first understand how turns and turn-taking are organized. There is still little research on social interaction in signed languages that adopts a conversation-analytic perspective. To date, we have been provided with some descriptions of how signers indicate their incipient speakership, or their willingness to keep a turn or assign a turn to a next participant. These descriptions do not necessarily follow the analytical mentality of conversation analysis. As a consequence of this, the notions of turn, TCU and TRP are still rarely discussed in detail in this field. Moreover, a most basic issue that has given rise to contradictory assumptions among researchers is the question of whether Sacks et al.'s (1974) turn-taking machinery is also valid for signed languages (e.g., Martinez, 1995; McIlvenny, 1995; Coates and Sutton-Spence, 2001; McCleary and Leite, 2013).

This study did not pursue the question of whether in signed languages there is indeed more overlapping “talk” as compared to spoken interaction. With the analytical focus chosen for this study, the present results provide the field with an initial systematic sketch of the *sequential environments* of overlaps in signed interaction. Using the conversation analytic tools for studying the organization of signed interaction data allowed us to see how participants themselves treat ongoing turns (as being complete or not) and overlaps (as being troublesome or not). The preceding analyses have shown that the vast majority of overlaps produced among four young female signers frequently occur within the sequential environment of possible completion (79.4% of all overlaps; Sections Simultaneous Signing at Places of Possible Completion, Simultaneous Signing after Places of Possible Completion, and Simultaneous Signing before Places of Possible Completion), i.e., they rarely occur in the midst of syntactic units (10.6% of all overlaps; 4.4). This shows that participants finely tune their turn beginnings to those places in the dynamics of interaction, where turns are *possibly complete*. Hence, the majority of overlaps results from the fact that incipient signers anticipate a turn-end and overlap it, and/or that current signers continue beyond a first possible completion. The findings therefore underpin the observed orderliness of overlapping talk in spoken language interaction (Jefferson, 1984, 1986; Schegloff, 2000). Adding to recent findings that demonstrated signers' orientation to precision-timing (De Vos et al., 2015) as well as practices for overlap resolution (McCleary and Leite, 2013), the current study further substantiates the claim that sign language users, too, orient to a turn-taking machinery based on the principle of “one-at-a-time.”

The data showed that signed overlap is not messy but organized. Admittedly, however, this does not disprove the assumption that signed interaction presents *more* simultaneous “talk” than spoken interaction, as proposed by Coates and Sutton-Spence (2001) or Lackner (2009). It might well be that signers start their overlaps in an organized manner, and then continue while simultaneously signing for longer stretches than it has been shown for spoken interaction. While I did not systematically investigate the length of the overlaps or overlap resolution practices, the description of what actions participants accomplish with the overlapping turns nevertheless provides us with some valuable insights. Major actions accomplished are acknowledgments, agreements and displays of understanding by means of short reformulations. Furthermore, overlapping turns were often observed in courses of action involving repair (repair initiations, repairs or change-of-state tokens). Within these actions, overlapping signing does however not imply that the “one-at-a-time” principle is invalid. In fact, participants orient to the fact that the default organization is *one-at-a-time* by keeping their turns short (as in acknowledgments and agreements), or by accomplishing actions that can reasonably overlap ongoing turns because of their urgency (repair initiations) or because of the interactional effects it thereby creates (e.g., strong disagreement). It seems therefore that the observations made on overlaps in spoken interaction are also applicable to the data under study in this article. Consequently, prolonged simultaneous signing and the existence of one-at-a-time principle are not necessarily mutually exclusive.

### Gradual turn endings and smooth turn transitions

An important finding of the present study is that ‘last item overlaps’ are scarce (cf. Section Overlapping a Current Signer's Last Item of a Unit). By contrast, participant B often overlaps a potentially last item of participant A who however continues past a possible completion (cf. Section Overlapping a Current Signer's Potentially Last Item of a Unit). This may raise the question of whether participants erroneously predict turn-endings. Another hypothesis would be that current signers who are confronted with overlapping signing by their co-participants continue their turns as a means of holding the floor. While the methodological tools of CA do not allow answering the first question, the sequential analyses of overlapping signing provide some evidence for answering the second one. The analyses have shown that at first places of possible completion, current signers' continuations regularly consist of one or more items, short add-ons. These added items often consist of non-lexical elements or signs that do not contribute substantially new information (*PALM-UP, PALM-DOWN, STOP, -H*). Moreover, after these add-ons, signers often finish their turns. This provides evidence, from a participants' perspective, that there is in fact no attempt to hold the floor. By contrast, on the basis of a fine-grained investigation of the collaborative work accomplished by the signer and his recipient's during possible turn transition, the present analyses suggest that participants deploy such short add-ons as an interactional resource for the management of turn transitions. Concretely, participants step out of their turns in a gradual and smooth manner.

These add-ons can occur after a pause (Section Simultaneous Signing after Places of Possible Completion) or latched to a first possible completion (Section Overlapping a Current Signer's New Unit at Places of Possible Completion; cf. also Section Overlapping a Current Signer's Last Item of a Unit). In the first case, the turn continuation can serve as a resource for treating a problem of recipiency, similarly to turn extensions in spoken language (e.g., Horlacher, 2007). In the second case however, participants may accomplish a slightly different interactional task. When participants continue their turns without any pause after possible completion, they may deal with the fact that neither of their co-participants launches into a turn that goes beyond a minimal response. Hence, the turn extension by a current signer is not oriented to an absence of response, but an absence of a turn-launching that will substantially add to the progression of the activity at hand (cf. e.g., excerpt 6). Concretely, the current signer therefore extends the turn boundary so as to permit a smooth transition without notable pauses between turns.

These observations provide us with some interesting insights regarding turn transition in signed languages. On the one hand, the present study supports that incipient signers orient to the end of strokes/independent holds as first possible transition places where they can launch a new turn (Groeber and Pochon-Berger, 2014; De Vos et al., 2015). On the other hand, as it has been shown for spoken languages, linguistic (syntactic and prosodic) units within a pragmatic context are “possible turns,” which can be further expanded with different types of constructions. Thus, turn boundaries are not fixed but flexible. Moreover, and this is a consequence of this first point, the transitions from one signer to the next are also not always clear-cut—within the transition space current and next signers overlap each other as a consequence of the fact that current signers regularly trail-off their turns, stepping out of them in a smooth manner. Consequently, an interesting hypothesis to pursue is the idea that a sense of more overlap in signed interaction may be due to the fact that participants regularly step out of turns in a gradual and smooth manner, rather than ending them with an abrupt retraction of the hands/forearms to rest position. This practice for designing turn transitions may be a specificity of signed languages, but more data has to be investigated to corroborate this idea.

Indeed, it must be emphasized that the present results are limited to one constellation of participants (four acquainted women) and a limited range of courses of action (question-answer sequences, storytellings). Whether the same type of turn transition can be found with other participants (male group, mixed group, unacquainted participants, L2 signers) is an open question. Moreover, as the excerpts in Sections Simultaneous Signing after Places of Possible Completion and Simultaneous Signing before Places of Possible Completion suggest, a systematic analysis of turn-endings that set a strong actional projection as compared to those without strong projection (e.g., questions vs. comments) will be necessary for a more detailed understanding of signers' management of turn-taking.

### Conflict of interest statement

The author declares that the research was conducted in the absence of any commercial or financial relationships that could be construed as a potential conflict of interest.

## Footnotes

^1^As Schegloff puts it, turns are “the organization unit which ‘houses’ grammatical units” (1996, p. 55).

^2^Signers make regular use of so-called productive signs, i.e., signs that are only partly conventionalized (they are semi-lexical). They constitute meaning by depicting situations and objects rather than denoting these. Their meanings depend heavily on context.

^3^It is however not clear whether she considers the retraction phase and the hold as still being part of the turn or not.

^4^Transition space is “(…) *the environment of a turn's possible completion, at which possible transition to a next speaker becomes relevant*.” (Schegloff et al., 1977, p. 366).

^5^The reason for this is that by the moment of analysis, I was more familiar with search functions implemented in ELAN than in *iLex*.

^6^A mouthing is the unvoiced articulation of a word or word part from the spoken language lexicon which is used in the hearing community surrounding the Deaf sign language community (Boyes Braem, 2001, p. 99). In our case, the mouthings are thus German words or word parts.

^7^The next turn proof procedure is a basic methodological tool within CA for the analysis of interaction within a participant's perspective. For the assignment of actions in interaction, analysts are held to ground their interpretation on how participants themselves understand an action. This necessitates an analysis over three turns, where A is accomplishing an action (e.g., information elicitation), B displays then what he understands this action to be by means of his response (e.g., providing information), and A then either confirms or disconfirms that B has understood adequately.

^8^Note that when A is reaching a possible syntactic completion, it is also possible that, precisely timed to the end of this completion two ‘new’ participants launch a turn, overlapping each other.

^9^Out of 47 annotated preparation phases, 43 instances are less than 250 ms. According to De Vos et al. (2015) this implies that these beginnings are precision-timed even if the preparation phase of participant B is launched after the stroke of participant A.

^10^This is not only the case for transitions between a current and a next signer, but also for cases where a current signer is reaching completion and two new signers launch into a turn.

^11^Also for this type of overlap it is possible that two new participants launch a turn after a pause that followed the completion of participant A.

^12^ABU is commonly used abbreviation for ‘Allgemeinbildender Unterricht’ (teaching in general knowledge).

^13^The mouthing –e- may be related to the German word “eben” (“there you have it”), which is also conventionally used with the sign *EBEN* consisting of a palm-up gesture.
